# Cardiovascular-Kidney-Metabolic Syndrome Stages via Intersectionality in the All of Us Program

**DOI:** 10.1093/geroni/igaf122.1736

**Published:** 2025-12-31

**Authors:** Xiang Qi, Junyu Sui, Bei Wu

**Affiliations:** New York University, New York, New York, United States; Arizona State University, Nursing and Health Innovation, Pheonix, Arizona, United States; NYU Shanghai, Shanghai, China (People’s Republic)

## Abstract

Cardiovascular-kidney-metabolic (CKM) syndrome, a newly defined, multistage disorder by the AHA, integrates cardiovascular, kidney, and metabolic dysfunction. While disparities in CKM stages across race/ethnicity, and sex are known, an intersectional lens is essential to uncover how these factors, combined with contextual deprivation, influence disease risk. This cross-sectional study analyzed differences in CKM stages among 126,892 adults aged 18-79 from the All of Us Research Program. Exposures included race/ethnicity (White, Black, Hispanic, Asian, Middle Eastern and North African, Other), sex, and contextual deprivation, measured via the Neighborhood Deprivation Index. CKM stages (0-4) were assessed using electronic health records, laboratory tests, and physical measurements, with stages 3-4 indicating advanced disease. Overall, 23.5% of participants had advanced CKM syndrome, specifically: stage 0 (10.0%), stage 1 (20.8%), stage 2 (45.7%), stage 3 (9.5%), and stage 4 (14.0%). Adjusted analyses revealed higher odds of advanced stages among Black (OR 1.97, 95% CI 1.90-2.03), Hispanic (OR 1.72, 95% CI 1.66-1.79), Native Hawaiian or Pacific Islander (OR 2.07, 95% CI 1.55-2.75), and Other race/ethnicity participants (OR 1.40, 95% CI 1.31-1.49) compared to White participants, while Asian and Middle Eastern or North African groups showed similar odds. Males (OR 1.58, 95% CI 1.55-1.62) and individuals in more deprived areas (OR 1.14, 95% CI 1.11-1.18) also faced elevated risk. Intersectional findings highlighted Black or Pacific Islander males in deprived contexts with the highest risk, contrasting with lower risk among Asian or Middle Eastern females in less deprived settings. These disparities emphasize the need for targeted, equity-focused interventions.

